# Collectanea

**Published:** 1829-08

**Authors:** 


### COLLECTANEA.

Floriferis ut apes in saltibus omnia libant,

Omnia nos, itidem, depascimur aurea dicta.

### ANATOMY.

*Rare and remarkable Anomaly of the Vascular System.*—This case was met with by Professor FRANCHE, and is related in the *Zeitschr. für Natur und Heilkunde*, for 1827.

The aorta was in its natural position, but the vena cava ascendens did not lie on the *right* side of the former vessel: it, on the contrary, ascended over the fourth, third, and second lumbar vertebræ, on the *left* side of the aorta. Opposite to the first lumbar vertebra, the vein crossed in front of the artery, immediately below the superior mesenteric. Having arrived on the right side of the aorta, the vein continued to ascend, as usual, towards the liver and diaphragm. At the point where the vena cava is formed by the union of the two iliacs, and where the aorta bifurcates, the iliac veins were for the most part covered by the iliac arteries; but the former were rather more to the *left* than the latter, corresponding thus with the anomaly of the principal trunks. The aorta was in this manner quite embraced by the vena cava opposite to the lumbar portion of the vertebral column.

The only similar case on record, we believe, is that noticed by MORGAGNI, De Sed. et Caus. Morb. lib. iv.—*American Jour. of Med. Sciences.*

*Insertion of the Umbilical Vein in the right Auricle of the Heart.* By Prof. MENDE.—The child who formed the subject of this case died immediately after its birth, without any known cause. It did not show any anomalous appearance externally; but, the vessels having been injected, a remarkable anomaly was discovered in the disposition of the umbilical vessels. The umbilical vein, instead of dividing into two branches to traverse the liver, continued in the form of one trunk, and ascended over the convex surface of the right lobe of that organ to the right auricle of the heart, where it terminated before and above the mouth of the inferior cava. The heart appeared to be pulled down by this insertion of the umbilical vein; its base was much inclined towards the right and towards the sternum; its position was, consequently, more transverse than ordinary. A single umbilical artery arose from the abdominal aorta, at its bifurcation, between the primitive iliacs; it passed on the left side of the urinary bladder, and continued its course to the umbilicus. No other anomaly was discovered in the abdominal or the thoracic viscera.—*Ibid.* from the *Bulletin des Sc. Méd.*

---

PHYSIOLOGY.

*State of the Heart during Pregnancy.*—It is asserted by M. LARCHER, in a paper in the *Archives Générales*, that in pregnant women the left ventricle becomes thicker, more firm, redder, and more active, than natural. This hypertrophy, he says, whether the cause or the effect of plethora, always imparts an energy to the circulation, which accounts for the vascular symptoms of pregnancy.—*Ibid.*

---

*Cyst near the Parotid Gland, containing a Fœtus.*—Professor RENNER, of Jena, relates, in the *Zeitschr. für die Organ. Physick.* for September 1827, his having found in a cow, which died of hectic fever, a cyst two inches in length at its greatest diameter, under the skin, behind the parotid gland, which contained the bones of a fœtus.—*Ibid.*

---

*Perspiration after Death.*—At a meeting of the Royal Academy of Medicine in August 1828, the secretary read a case which came under the observation of M. SPERANZA, clinical professor at Parma. The subject was a woman of Mantua, who died on the fourth day after an attack of encephalitis. Examined twelve hours after death, the body was still warm and covered with a profuse perspiration, which, as often as it was wiped off, returned again. This phenomenon continued during twenty-four hours. M. FONTANEILLE, by whom the case was addressed to the Academy, suggests, in explanation of this occurrence, that the cutaneous capillary vessels still retained a remnant of the vitality proper to them after the cessation of general vitality.—*Ibid.* from the *Archives Générales.*

---

PATHOLOGY.

*Medical Report of the House of Recovery and Fever Hospital, Cork street, Dublin.* By JOHN O'REARDON, M.D.

ALTHOUGH we do not deem it necessary to give the whole of this report, there are many pathological and practical remarks contained in it, which result from the extensive experience of Dr. O'Reardon, that it would be improper to pass over. We shall select those which appear the most useful and interesting, either because they confirm opinions previously entertained, or from their suggesting any novel views.

The chief visceral accompaniments of fever in spring were cephalæa, pneumonia, catarrh, rheumatism, abdominal inflammation, and sub-inflammation; dysentery, and a few cases of erysipelas of the face and head. The last-mentioned disorder, even in its more formidable shape, "I generally or almost universally cure," says Dr. O'R., "by one, and occasionally two, moderate arm bleedings, followed by the use of three or four drachms of cinchona, given in two portions daily, the bowels being at the same time kept free by any of the ordinary cathartics. I find this practice uniformly succeed in cases of alarming tumefaction of the face, eyelids, and integuments of the head, and delirium; although in many of these instances there be a smallness, and a rather discouraging deficiency of strength of the pulse."

Dr. O'Reardon had under his care two patients, one a man and the other a woman, each severely afflicted with a complication of dysentery and melæna. In addition to the usual well-known dysenteric stools, both these persons frequently voided large heavy clots of coagulated blackish blood, of most offensive odour.

"These repeated losses rendered the lips and faces of the patients completely blanched, and caused considerable languor. The skin was in both instances hot, and the pulse was quick and small. I prescribed for each a mixture as follows: ℞ Olei Ricini ʒv. vi.; Ol. Terebinth. rectificati ʒiij. iv.; Mucilaginis Acaciæ ℥iss.; Tincturæ Opii ℈i.; Syrupi ℥ss. Fiat mistura, cujus sumatur pars quarta tertiis horis ad quartam vicem. The prescription was the same for both patients, except that the mixture directed for the man contained a drachm of the Oleum Ricini and a drachm of Oleum Terebinthinæ more than that for the woman. I repeated this remedy daily for three or four days, and again directed the same during two days more with diminished quantities of Oleum Terebinthinæ and Tinctura Opii. By such treatment the melæna was in each patient completely cured, and the dysentery was much mitigated. The latter soon afterwards yielded to two or three doses of Oleum Ricini."

Whatever the sticklers for nosological divisions and refinement may think of the following remarks, they are such as every practical observer will recognise as arising from observation at the bedside of the patient.

"The divisions of fever designated in our classic medical works by the appellations of Synocha, Synochus, Typhus mitior, Typhus gravior or putridus, Febris biliosa, Febris nervosa, and Febris maligna; and which Pinel terms Fièvre anquiotenique ou inflammatoire, Fièvre meningo-gastrique ou bilieuse, Fièvre adenomeningée ou muqueuse, Fièvre adynamique ou putride, Fièvre ataxique ou maligne: all these divisions are, I venture to say, mere variations of one genus, that is, of the above-described general sub-inflammatory condition, constituting fever: varieties produced by the diet, occupation, locality, temperament, habit of body, state of mind, or nervous condition of the patient; or by an alteration in his biliary or other secretions, or by the period of the fever, or its treatment; or by the season of the year, the climate, or constitution of the atmosphere.

"I must likewise include, as species belonging to the forementioned genus, the Peripneumonia biliosa, Peripneumonia putrida, Peripneumonia maligna, Catarrhus biliosus, Catarrhus putridus, Catarrhus malignus, of some authors; as, in tracing the histories of these diseases in the works of some of the physicians who record them, I find them to be the ordinary kinds of fever, combined, some with catarrh, some with pneumonia, and others with abdominal ailment.

"The varieties of fever in different countries, and in different parts of the same country, are principally to be set to the account of diet, locality, air, and climate. The basis or general plan of treatment ought, I conceive, to be similar in all countries; save that their varieties and changes demand corresponding modifications in the treatment. In unfavorable situations, and very hot oppressive climates, the progress of fever through its stages is rapid, and must necessarily be met by appropriate activity, vigilance, and discernment.

"The above view of fever, which in my mind is that of nature, removes that obscurity and confusion which long prevailed, and in some places still exists, in the management of fever. Though comprehensive, it does not confound any diseases which are naturally separate. It admits and requires the utmost attention in distinguishing the varieties and successive changes of fever, and in discriminating the character of each disordered viscus. It indicates a rational, clear, effective, and tolerably uniform line of practice in the treatment of fevers, and their manifold complications; a practice which the best modern physicians are, for these many years back, gradually adopting, as if it were by common consent.

"This practice consists mainly in the antiphlogistic plan of treatment, modified and varied according to circumstances, and especially according to the character, grade, and period of the fever, its complications, and the patient's pulse and strength.

"Bilious and hepatic symptoms are to be subdued by well-known appropriate remedies, similar to those prescribed for the hospital nurse, whose hepatic disease and recovery are noticed. These are remedies which, so far from being opposed to, are for the greater part in unison with the forementioned antiphlogistic plan. This plan, based, it is presumed, on sound principles, is extensive and varied in its application. It admits of the mildest practice in mild cases, as well as of the most active and efficient treatment in severe cases. It is suitable not only to the diseases above mentioned, but is also applicable to the exanthemata, or eruptive fevers, which require to be treated pretty much on the same principle with common fever and its modifications; but, in the exanthemata, we must be cautious to prevent the disappearance of the eruption before the proper period, and we must be ready to obviate or remedy visceral determinations."

Whatever may be the appearance of fever in its commencement, and by whatsoever title it may be denominated by nosologists, whether synocha, synochus, or typhus, in its advanced state it becomes occasionally marked by adynamic spasmodic nervous symptoms, with a black or blackish tongue, and a very peculiar alteration of countenance; symptoms which supervene on, or take place of, the acute sub-inflammatory or inflammatory stages.

"Under these circumstances other indications arise, which call forth the application of the whole medical mind. The *methodus antiphlogistica* is to be employed not only in a very mitigated manner, but it must be combined with, and often superseded by, antispasmodic, antinervous, diffusively stimulating, and tonic remedies. As, in the class of tonic medicines, cinchona holds a prominent station, I think it right to observe that this article is scarcely ever useful, and is often injurious, in continued fever, during the existence of a hot skin, a quick pulse, and a loaded tongue; cases of gangrene excepted, where indeed the temperature is in general any thing but high, and where cinchona, aided by camphorated mixture, good wine, and a suitable position of the body, is an extremely valuable curative agent."

Dr. O'Reardon gives, a few minutes before the time that the paroxysm of ague is likely to occur, "a draught composed of three or four drachms of Ether Sulphuricus, from fifteen to twenty-five drops of tincture of opium, two ounces of water, and two or three drachms of simple syrup." The dose of ether here prescribed is unusually large, and we think would scarcely be borne by many patients. We have in many instances put off the fit of ague by a larger dose of opium, without the combination of ether. Dr. O'R. has known some intermittents recover well under very mild treatment, such as an emetic a short time before the rigor, and afterwards, during the intermissions, Infusio Anthemidis and Vin. Absynth. "On the other hand, I have seen not a few women fall victims to trivial and inert practice, and to the erroneous prejudices of a late celebrated Parisian physician against the employment of cinchona in ordinary intermittent cases. The result was that some agues, not originally dangerous, became more frequent and severe, caused considerable debility, and at length induced dropsy and diseased alterations of the spleen, liver, pancreas, and even kidneys, especially of the first-mentioned viscus."

---

*Hydrophobia.*—M. Husson lately showed, at the Academy of Medicine, a part of the tongue of a child, eleven years of age, who had been bitten on the cheek by a rabid dog. Nineteen days afterwards, she was attacked by hydrophobia, and death occurred two days after the invasion of the disease. Upon the mucous membrane of the portion of the tongue which was exhibited, there were about thirty pustules, very close together, of a flat shape, with a depression in the centre. They were thought to resemble the pustules of variola.—*Arch. Gen.* May 1829.

---

*A Case of Perforation of the Stomach and Œsophagus, with brief Remarks.* By Marshall Hall, m.d. f.r.s.e. &c.

The little girl, whose case I am about to describe, had been subject, from a very early period after its birth, to attacks of bronchitis.

Early in April it became affected with pertussis. The symptoms of bronchial and pulmonary inflammation called for the abstraction of blood; and three, and then two, leeches were applied to the chest on two successive days, with other remedies usual in such cases. This was followed by exhaustion with reaction, the countenance varying, being sometimes pallid and cold, and sometimes flushed; and the pulse frequent and jerking. Soon after the second application of leeches, there were also frequent fits of convulsion, for which a cold lotion was applied to the head; and the warm bath was used frequently. The Hydrargyrum cum Creta was administered, with a mild nutritious diet. There was no sickness, no diarrhœa.

After a variable state of things, this little patient sank and expired, having lingered eight days.

Permission could not be obtained to inspect the body until the fifth day after death. The morbid appearances were then carefully noticed by Mr. R. Welbank and myself.

The general surface was extremely pallid, but there was little or no emaciation.

The bronchiæ were clogged with mucus, and the lowest lobe of each lung was hepatised.

On looking into the right cavity of the thorax, a small portion of venous blood was observed. The source of this was carefully traced. A small part of the pleura immediately adjacent and above this spot, extending upwards over the convex surface of the vertebræ, was found perfectly removed by erosion; the subjacent veins had been opened by the same process, and their blood had escaped; the nerves were left entire, as it were beautifully dissected. Proceeding with the examination, there was found, at a part which corresponded with these appearances, an opening that penetrated into the œsophagus; and through this opening a portion of the contents of the stomach flowed, on raising this organ. At the same moment the rest of the contents of the stomach escaped into the abdomen, through a large orifice at its most dependent part.

On further examination of the state of the œsophagus and stomach, the mucous membrane was found uniformly reduced to a gelatinous mass; the textures constituting the former were pierced by an irregular opening, of a size less than that of a pea; the peritoneum covering the latter was destroyed to a considerable extent. But there were no appearances of disease about the edges of either orifice.

The head was not examined. The other viscera presented no unnatural appearances.

The case thus briefly detailed leads to some remarks of great interest:

1. It cannot be doubted that in this case the perforations of the œsophagus and of the stomach resulted from the action of the gastric juices after death. This appears to be proved by the eroded state of the adjacent parts. This fact may, therefore, be regarded as established by the present and similar cases.

2. It is equally certain that there is one special disease or disorder of infants which leads to similar results, as stated in the interesting and valuable paper of Dr. John Gairdner, in the Transactions of the Edinburgh Medico-Chirurgical Society, vol. i. p. 311.

3. It is a point of the utmost importance to state, in the account of post-mortem appearances, at what precise period after death the examination was made; and it might be useful sometimes to make the examination at two distinct periods, taking care not to disturb the parts at the first. It is quite plain that, had the parents of the little girl whose case has been given, earlier consented to an examination of it, some of the appearances which have been described would not have been observed.

4. It would be interesting to make a series of observations on rabbits and other animals, with a view of determining the circumstances which favor or oppose the erosion of the stomach by the gastric juice. The observations made by Dr. W. Philip, in the third edition of his singularly admirable work on the Vital Functions, pp. 131-2, appear to be too general on this point.

5. We might possibly employ the gastric juice in the minute dissection of the nerves, since this texture appears to resist the action of this agent, whilst that of the other parts is destroyed by it. The fact itself is mentioned by

M. Cruveilhier, in his Medecine Pratique, Cahier i. p. 143.—*Edinburgh Med. and Surg. Journal.*

---

### PRACTICAL MEDICINE.

*Combination of Lactuca Sylvestris and Digitalis in the Treatment of Hydrothorax.*—We are indebted to Dr. Teel, of Aurich, for this mode of practice. M. Brosius has applied it in twelve "inveterate" cases of hydrothorax, and has much confidence in its efficacy. Although but two of these cases were radically cured, the symptoms of eight others were very materially relieved. In two patients only the remedy appeared to exert no beneficial influence; and in these instances the fact was corroborated, that, if the proposed remedy does not relieve during the first days of the disease, no advantage is to be expected from its continuance.

One of the complete cures surpassed every previous hope. The patient was a woman seventy-four years of age. She took four grains of the Extr. Lactuc. with one grain of the powder of Digitalis in a dose, every two hours. After the fourth dose the symptoms were much relieved; after the sixth, they had disappeared; and at the end of three days, during which time the patient had taken in all eighteen doses, a strong infusion of digitalis was prescribed. The cure was completed by light bitters.

In one case in which this combination only acted as a palliative, the patient was relieved in five attacks, in each of which the face, hands, and feet were œdematous. The sixth attack proved fatal.—*Journ. der Prak. Heilkunde.*

---

*On the Employment of the Extract of Male Fernroot for the removal of Tænia.*—Dr. J. J. Ebers has published eight cases in which the above medicine has been followed by complete success in the removal of tænia. The dose prescribed by the Doctor is from eighteen to twenty-four grains, administered at two doses, under the form of pills. He has repeated this two or three times occasionally, though in general one dose has been sufficient to cure the patient. He generally orders a purgative to be taken on the following day, which produces the evacuation of the worm, for the extract appears rather to have the property of killing the worm than expelling it.

Dr. Ebers draws the following conclusions from his experience:

1. The extract of male fernroot is one of the surest means that can be employed against the tapeworm.
2. It generally kills the worm speedily, and thus favors its expulsion from the body.
3. It acts as a specific.
4. It does not expel the tænia in a ball or mass, as other anthelmentics.
5. This medicine acts usually in a mild manner, and without producing any severe symptom: once only it produced some severe effects in a female who had not the tapeworm.
6. It also expels ascarides, but with this difference, that it does not kill them.—*Journ. de Chimie Med.*

---

*Diagnosis of acute Idiopathic Inflammation of the Heart.*—Dr. Heim, of Berlin, has laid down the following symptoms as diagnostic of this formidable malady:

The disease begins with shivering and trembling of the whole body, and intermittent chill, which latter, when present, may continue during twenty-four hours, but is followed by very little or no heat. There is no acute stitch felt, but pains in the heart precede the distinct attack for twenty-four hours. Sometimes the disease comes on suddenly. The patient has no cough: if in any instance cough occurs, it is entirely dry, neither mucus nor blood being expectorated. In the commencement the patient has the greatest anguish and the most agonizing pain, not in the chest generally, but immediately in the heart itself. The patient shrieks out, and is not quiet more than a second, repeating with great force the same word three or four times to express his most distressing sensations. He does not lie still an instant, but tosses himself about in bed like one half distracted, while his arms and head are in continued motion. The patient presses upon the region of the heart; and, if pressure be made by a by-stander, he impetuously demands that it shall be increased. The countenance is always extremely pale. The chest is elevated, and the head, which is in perpetual motion, is thrown more backwards. The face and hands are quite cold. Before each necessary bleeding, the pulse is throughout not to be felt. The patient feels every movement of the heart, and even complains of its painful throbbing; yet, when the physician applies his hand to the chest, he cannot discover the least irregularity. The stronger the pulsation of the heart is, the greater is the pain felt in the organ: it seems to the patient to strike upon a wounded spot. He is nauseated, but does not vomit; the greater his thirst is, so much the more does he refuse to drink, even when a glass of water is held to his mouth. He is very loquacious, even when otherwise naturally silent: we might also say physically, what the Scriptures assert morally, that "out of the fulness of the heart the mouth speaketh." Fainting and delirium are not uncommon.

A copious bleeding is followed by so great an alleviation of anguish and pain, which lasts for several hours, that the patient thinks himself quite cured. But the symptoms suddenly return again after the cessation. The anguish and pain is increased by warm applications to the chest, and the patient throws them off. If the treatment be neglected, or improper, the patient dies either in consequence of polypous formations on the internal or external surface of the heart, as well as on the internal surface of the pericardium, or with adhesions of the heart to this membrane, and effusions of pus or water into the cavity of the pericardium.

In the *Literarische Annalen der Gesammten Heilkunde,* Dr. A. H. Krause, of Berlin, also relates a case of acute idiopathic inflammation of the heart. In the case witnessed by Dr. K. the above symptoms were well marked. Free bleeding, digitalis, and other obvious and appropriate treatment, restored the patient to health in less than a fortnight.

---

*Observations on Spasm or Cramp of the Stomach; with Cases and Dissections.* By John Macfarlane, m.d.

Spasm of the stomach, although often sudden in its attack, urgent in its symptoms, and alarming in its appearance, has been either altogether overlooked by the majority of authors, or noticed only in the most cursory manner, as an occasional attendant on dyspepsia. It is, however, an important, frequently occurring, dangerous, and sometimes fatal, variety of stomachic disease. Its symptoms are in general well-marked and diagnostic. The treatment requires to be prompt, powerful, and peculiar; and although in several cases it may be connected with a previously existing derangement in the functions of the affected organ, yet in others, and these by no means rare, it originates suddenly from distant irritation, or without any previous morbid indication.

When spasm affects the stomach, there is the most acute pain, with a feeling of rigid contraction, violent twisting or tearing in the epigastrium, soon followed by painful and interrupted breathing, difficult articulation, pallid countenance; small, hurried, and contracted pulse; and occasionally with coldness of the extremities, and rigid contraction of the recti abdominis and gastrocnemii muscles.

In severe forms of the disease, the patient usually complains of a sensation of rigid contraction, or drawing together, in the epigastric region, occasioned by the inordinate contraction of the muscular coat of the stomach, and occasionally producing a hard circumscribed tumor, perceptible to touch. When, however, the abdominal muscles participate in the spasm, the tension and inequality of surface produced by the morbid contraction of the recti abdominis effectually prevent the discovery of this tumor. The diaphragm, it is presumed, very soon sympathises with this state of the stomach, and becomes also spasmodically affected, as the short, interrupted, and highly distressed respiration, and the difficult articulation, evidently show. Indeed, every person who has seen a violent attack of this complaint must have observed the change in the respiration which takes place at the height of the paroxysm; the difficulty, and often the impossibility, of performing inspiration and expectoration in an unobstructed manner, and the half-suppressed cries or moans which the patient utters, apparently occasioned by the rigidly contracted diaphragm, remaining as an almost immoveable partition between the thorax and abdomen. If the hand is applied either to the thorax or epigastrium, we can seldom discover the alternate elevations and depressions of these parts indicative of a natural state of breathing.

With respect to the causes of the disease, the author has seen several instances where it was produced by great mental anxiety. In some cases, where a strong disgust or antipathy exists to certain dietetic articles, any attempt to eat them, or even simply naming them to the patient, has been followed by severe spasmodic affections of the stomach. But the cases are, however, far more numerous in which the disease is produced, not through the influence of the imagination, but from the introduction into the stomach of some substance, which, from peculiar idiosyncrasy, acts on this organ as a morbid irritant. In addition to these exciting causes may be ranked sudden exposure to cold, drinking cold liquids while the body is heated, coldness of the lower extremities, intemperance, &c.

"Females are more subject to this disease than males, in the proportion of two and a half to one. Accordingly, of thirty-six cases which I have seen, twenty-six occurred in females, and ten in males; and, in twelve of these, no affection of the stomach, or other predisposing cause, could be discovered. Irritation in the uterus is also said to be a frequent cause of spasm of the stomach. Cullen says, that 'the ordinary flow of the menstrual discharge retarded, or totally suppressed, affects the stomach, and disposes it to be affected more readily with spasm.'"

When long continued, spasm of the stomach is apt to induce inflammation of this organ. The occurrence of violent hæmatemesis during a paroxysm of spasm of the stomach, probably occasioned by a partial laceration of the internal coat of that viscus, is illustrated by a case, in which the patient recovered.

An interesting case is related where death took place in little more than an hour from the commencement of the spasm, and where, although the body was not allowed to be examined, the author thinks the fatal event was produced by laceration of the stomach from the violence of the spasms.

In another instance, where the symptoms were well marked, and the history of which is given, a lacerated opening was found in the stomach on dissection, without the slightest vestige of organic disease, of gangrene, erosion, or ulceration.

The disease may prove fatal without inducing any lesion of the stomach, and an instance of this kind is detailed where, on dissection, the only morbid appearance that could be discovered by the most accurate investigation was general softening of the cerebellum, with vascular turgescence in the base of the brain.

In the treatment of spasm of the stomach, where we find it occurring in individuals whose general health has been impaired by confinement or sedentary employments, or who have suffered from anxiety, fatigue, or exhaustion, and who are free from stomachic ailments, the author has found the paroxysms frequently subdued by a drachm of sulphuric æther with fifty drops of laudanum, its good effects being sometimes instantaneous; while in other cases the dose required to be repeated two, three, or even four times, before relaxation of the spasm was effected. In a few other cases the same decisive results were obtained, although the medicine was speedily rejected by vomiting. "On one occasion, (says the author,) when I was about to operate on a woman for strangulated hernia, the husband, a stout robust man, on account of anxiety for his wife, was suddenly seized with nausea and slight vomiting, followed by excruciating pain in the region of the stomach, and the other symptoms of violent spasm. A bladder containing pounded ice, which had been applied to the hernia, was laid over the epigastrium, and with the happiest effects; for in less than five minutes the pain was removed. This application is much recommended by M. Barras in neuralgia of the stomach; but I have had no other opportunity of trying its efficacy." When the attack is produced by the introduction into the stomach of some morbid irritant, the speediest relief will be obtained by the exhibition of an emetic.

"I have in two cases seen the most marked advantage from venesection; and that when, from the aspect of the patients, the cold clammy state of the skin, and the feebleness of the pulse, the reverse of this treatment seemed to be indicated."

When the recurrence of this disease is connected with functional derangement of the stomach, much benefit is found from small doses of quina, but especially from the use of the subnitrate of bismuth. When the attack is excited by depraved intestinal secretions, or by constipation, which frequently happens, more benefit is to be derived from mild laxatives and alteratives, than from strong or drastic purges. The diet should, of course, be strictly attended to, and such articles selected as are light and of easy digestion; for, when the stomach is much stimulated, either by the quantity or quality of the food, spasmodic excitement, more or less powerful, is not unfrequently produced.—*Glasgow Med. Journal.*

*New Mode of administering Quinquina.*—Dr. P. Richet, of Metz, relates, in his Thesis presented to the Faculty of Medicine of Strasbourg, four cases of facial neuralgia, which, after resisting the ordinary treatment, yielded to the administration of powdered quinquina one grain, and snuff two grains, mixed, and used as snuff. The above dose was always sufficient, and in from two to three days the patients were cured as if by enchantment.

---

*Treatment of Nymphomania.*—Dr. Ozanam, of Lyons, communicated to the Royal Academy of Medicine the cure of a case of nymphomania, by touching the swollen genital parts with a solution of four grains of nitrate of silver in an ounce of water. A slight eschar resulted from this application, and the sensibility of the parts were decreased; and in four days, by the application of this mild caustic, repeated twice a day, the patient was cured.—*Journal Général de Médecine.*

---

*On the Vinous Tincture of the Seeds of Colchicum Autumnale.*—Professor Chelius has ascertained that, during the use of the above medicine, the proportion of uric acid in the urine is very much augmented, and he attributes to this fact the benefits which it produces in rheumatism and arthritis. Dr. C. has employed the vinous tincture with success, both in acute and chronic arthritis; and with advantage also in sciatica, rheumatic ophthalmia, articular dropsies, and some cases of paralysis of the inferior extremities, not produced by an arthritic cause. Dr. C. commences with a dose of from twenty to thirty drops, and gradually and cautiously increases it, till signs of gastric irritation manifest themselves. He has never seen it produce ill effects, but it must be given with caution: he thinks the dose given by the English practitioners too large.—*Heidelberg Klinische Annalen*, tome iii.

---

### SURGERY.

*Pannus successfully excised.* By Professor Graefe.—A man, aged forty-five years, blind of both eyes, in consequence of the whole of the conjunctiva of the cornea and sclerotica of both eyes being changed into a vascular thick membrane, having been treated, without benefit, by partial excisions and topical applications, Professor Graëfe resolved to excise it. Accordingly, having raised this membrane, near the cornea, with forceps, it was dissected with scissors from half the cornea of each eye. As soon as the inflammation produced by this operation had disappeared, the membrane was excised from the other half of the cornea. Some days after, the rest of the membrane was removed in the same way. Vision was restored, and the patient was entirely cured by solution of opium, applied to the eye with a brush.

M. Graëfe cites this case to show, that the excision of this description of pannus is not effectual, unless performed part at a time, and at periods sufficiently near, to prevent the membrane being reproduced, and with the precaution of not excising too much at a time, lest dangerous irritation should be produced.—*Institut de Clin. Chirur. et Oph. de l'Univer. de Berlin.*

---

*On Ligatures of Ruptured Arteries complicated with Fractures or Gun-shot Wounds.*—The rupture of a principal artery of a limb, in fractures and gunshot wounds, has been supposed to require the amputation of the limb. M. Dupuytren has tried, in two cases, the tying the artery, some distance from the injury, as in the operation for aneurism, and with the happiest success. Professor Delpech, of Montpelier, has also performed it with advantage in one case. M. Dupuytren has published an interesting memoir on the subject in the fifth volume of the Repertoire d'Anatomie: he thinks that many limbs may be saved by this operation.

---

### MIDWIFERY.

*On the dangerous Effects of Secale Cornutum.* By R. M. Huston, M.D. (*North American Med. and Surg. Journal,* January 1829.)

Our own experience in the use of this now very general remedy, is much too limited to justify us in drawing any positive conclusions as to its merit. The opinion we have formed from its employment in a few cases is, that its power of increasing uterine action is very uncertain; but we have had no reason to suspect that it has produced any ill effects either upon the mother or the infant. As we have made frequent extracts from the American Journals in praise of the ergot, we deem it incumbent upon us to take from the same source some remarks in proof of its dangerous effects, which are deserving the deliberate attention of practitioners of midwifery. The paper from which we select the following observations was read before the College of Physicians, by Dr. Huston.

As far back as the fifteenth century, the ergot, or spurred rye, was known to exert a very powerful influence upon the human system, as well as upon that of brutes; for we find the production of epidemics, mortification of the limbs, convulsions, &c. was at that time, and subsequently, unhesitatingly ascribed to it. It does not appear, however, to have been used in regular practice until its recent introduction by Dr. Stearns, of New York. Dr. H. remarks that, in Philadelphia, a very large proportion of the tedious, difficult, and unfortunate cases in midwifery occur among those women who are remarkable for their great muscular strength, and who possess a tone and rigidity of every part that nearly bids defiance to all the means we can employ to overcome it; and yet such women have weak, trifling, and inefficient labour pains; nor do they alter until, by active depletory means, or until they are worn out by days and nights of restlessness, anxiety, and fatigue, the system loses great part of its tone. On the contrary, the emaciated, consumptive, or the spare and delicate city lady, has often the most rapid and efficient labour. Nor is the speedy termination of such cases solely from the want of resistance. The uterine contractions are really powerful.

From these facts we learn the necessity there is for the temporary suspension, to a considerable extent, of the tonicity of the parts concerned in parturition, in order to ensure a prompt delivery. Not only that we may avoid delay from the resistance which would otherwise be afforded by that state of the parts, but also the constant and long-continued pressure which would thereby be exerted upon the fœtus, cord, and placenta; and that the whole nervous or motive energy of the parts may be concentrated in the parturient paroxysms, without any drawback from the co-existence of a different and antagonizing action. A moment's reflection upon the known effects of ergot will enable us to judge how far it is calculated to answer these indications.

The property for which it is most remarkable is, that of increasing the tonic action of the uterus. After its exhibition, if the hand be placed over the uterus, it will be found to be constantly hard, owing to a *tetanic* or permanent contraction of its fibres; whereas, in natural cases, the uterus hardens under the hand during a pain, and relaxes again as soon as it goes off. The mode in which the ergot acts is also shown by the scanty discharge of blood which takes place at the time of parturition, even in cases where there is great constitutional predisposition to hemorrhage. If, then, it be true that the tonic contraction of the soft parts concerned in parturition, more especially the uterus, is neither a natural nor, under ordinary circumstances, a desirable condition until after the delivery of the child; and if it be the principal tendency of ergot to excite or produce this state of the parts, the danger of administering it, even under the circumstances usually indicated by authors, must be sufficiently apparent.

From his own experience, Dr. H. is convinced that the ergot is a most dangerous and destructive drug. He makes this declaration under the fullest sense of the great responsibility which every man ought to feel when giving testimony upon a subject of such importance, and from a conviction that the medicine is now doing incalculable mischief.

It is stated in a note, that in no other place in the United States, nor perhaps in the world, is ergot so much praised, and by such distinguished men, as in Philadelphia; and in no other of the principal towns in the United States, judging from their bills of mortality, is the number of stillborn children in so large a proportion to the whole number of deaths. This item in the bills of mortality has become so glaring as to attract the notice of the newspapers.

In the course of the last thirteen years, Dr. H. has used the ergot in a large number of cases. He has never given it to a woman in labour, excepting when the pains were trifling and ineffectual; nor then, until he had satisfactorily ascertained that the head was well situated, the pelvis of good dimensions, the os uteri thoroughly dilated, and the external parts in a soft and yielding condition. In several instances he was compelled to use the forceps after the complete failure of the medicine; and, when it succeeded, the children were stillborn in a proportion which shocked his feelings. As a proof that the same results have happened to others, the following evidence is adduced.

Of seven cases reported by Dr. Church, in his Essay on Ergot, there were but two liveborn children. It is true he does not attribute the deaths to the medicine, but the fact is striking.

Dr. Halcombe, in a letter to Dr. Dewees, says, "For some time I used the scruple doses, or corresponding doses of the decoction, which I am afraid are every where yet too common; but soon abandoned this practice, *in consequence of several fatal demonstrations of its impropriety*." The dose, of which the ill effects are here so ambiguously spoken of, is that which is recommended by nearly every writer on the subject. Indeed, so decidedly pernicious has it been in the hands of some, that, unable to account for it in any other way, they have supposed it actually to have poisoned the child in utero; and Dr. Halcombe expresses his firm belief, in the letter already referred to, *from what he has seen and heard*, that more children have already perished by the injudicious use of ergot, during the few years which have followed its introduction into the practice of this country, than have been sacrificed by the unwarrantable use of the crotchet for a century past! It is no set-off to say that Dr. H. speaks only of its *injudicious* use; for that which he mainly combats as injudicious is scruple doses, the very thing which our most judicious writers recommend.

A reference to the paper of Dr. WARD, of New Jersey, will show that the practice has been but little, if at all, more successful in his hands.

In a paper by Dr. CHARLES HALL, of St. Alban's, Vermont, published in the American Medical Review, and republished in France, we have the following observations: "Although, in most cases of real labour, the child is forcibly propelled into the world by the aid of this article, there are, nevertheless, instances in which its power is not exerted to this end. Instead of that powerful unceasing increase in the pains of labour, which so astonishingly expedites the expulsion of the fœtus, *it sometimes excites constant distress of a general nature, without any apparent influence on the efforts of labour.* In cases where it does not favor immediate expulsion, it seems to have *a fatal tendency on the child; for in such cases the child is generally stillborn.*" He adds, "I have frequently had recourse to it; and have learned, not only from my own experience but from that of others, that it does not always increase the pains of travail; *that it is hazardous under any circumstances, and occasionally produces fatal effects.*"

Dr. WM. MOORE, of New York, whom Dr. Hosack speaks of as an eminent and judicious practitioner, says, "IT APPEARS TO BE INJURIOUS TO THE CHILD AT ALL TIMES; for, in every case in which I have seen it exhibited, *the child has been stillborn; and, in the greater part of them, it was not possible to restore it to life.*"

Dr. HOSACK, in his letter to Dr. HAMILTON, of Edinburgh, on this subject, remarks that, in three cases in which he gave it, "although no evidence existed, previous to the use of the medicine, that the fœtus was not living, *in every case in which it was administered the child was stillborn.*" In fact, so convinced is Dr. Hosack of this general consequence from the use of this article, that in the same letter he uses this emphatic language: "The ergot has been called in some of the books, from its effects in hastening labour, the *pulvis ad partum:* as it regards the child, it may, with almost equal truth, be called the *pulvis ad mortem;* for I believe its operation, when sufficient to expel the child, in cases where nature is, alone, unequal to the task, is to produce so violent a contraction of the womb, and consequent convolution and compression of the uterine vessels, as very much to impede, if not totally to interrupt, the circulation between the mother and the child.

Dr. Huston suggests, also, that some mischief may be caused by compression of the child's brain for a longer than ordinary period, from the premature contraction of the uterus not suffering the head to recede at regular and ordinary intervals, so as to relieve this condition by the natural elasticity of the parts.

Dr. Huston is aware that the position assumed of the fatal effects of ergot, is in opposition to the opinions and experience of some of the most eminent of the profession. This discrepancy may, he says, perhaps, be in part accounted for from their patients being in that rank of life where every comfort can be procured, and where accidents, privations, excessive fatigue, and the many untoward circumstances which give rise to bad cases in the inferior ranks of life, are seldom felt. Among that class of patients, from their general habits of ease and indulgence, there is necessarily a softness and flaccidity of the muscular system, which may admit, or even demand, the use of an article which so powerfully excites the tonic action of the uterus, and which, from that very property, would be inapplicable to general practice.

Dr. H. is aware that it is admitted by the admirers of ergot that its improper use is doing incalculable mischief: this being conceded on all hands, and being reprobated by all, constitutes no part of the object of his paper, which is meant to warn the profession of the danger there is in using the article under *all* the restrictions usually imposed. He does not assert it will always prove fatal, as apprehended by Dr. Moore. He knows to the contrary: but that it will very often, with all the prudence and circumspection that can be used. We presume these remarks are meant to refer to the infant, and not to the mother.

Of ergot as an emmenagogue Dr. H. has no experience, but its utility in menorrhagia he has experienced ; and this fact seems, he thinks, to contraindicate it in the former capacity.

The opinions of Dr. H., and the other practitioners whose evidence he brings forward to prove the dangerous effects of the ergot, are decidedly in opposition to the sentiments of the various members of the profession who have given the remedy extensive trials in this country. We have heard of a few cases in which it was doubtful whether the child had not suffered from its employment; and in one case, where it was given, we believe, to arrest hemorrhage after abortion, the woman suffered severely from constitutional disturbance, which could not, however, with any certainty, be attributed to the ergot. If every accoucheur who employs this remedy will *candidly* and *impartially* state the effects that it produces both upon the mother and child, and at the same time *distinctly* describe the *particular circumstances of each case* in which he has exhibited it, we shall no longer have to lament so distressing a difference of opinion upon a subject of such immense importance ; for a body of evidence would thus be procured from which correct inferences may assuredly be derived.

---

